# Immediate effects of dynamic- and static-based custom insoles on structural alignment, pressure distribution and subjective comfort in paediatric flexible flatfoot

**DOI:** 10.3389/fbioe.2026.1927139

**Published:** 2026-10-12

**Authors:** Sin-Hang Luk, Kit-Lun Yick, Chu-Po Ho, Joanne Yip

**Affiliations:** 1 School of Fashion and Textiles, The Hong Kong Polytechnic University, Hong Kong, Hong Kong SAR, China; 2 Laboratory for Artificial Intelligence in Design, New Territories, Hong Kong SAR, China

**Keywords:** insole design, insole geometry, paediatric flatfoot, plantar pressure, rearfoot biomechanics

## Abstract

**Background:**

Customised insoles for paediatric flexible flatfoot are typically designed using static foot postures, which may fail to reflect the dynamic mechanical demands of gait. This study explored an alternative method, evaluating whether dynamically modelled insoles, captured during active locomotion, can effectively provide optimized kinematic control and plantar load distribution while maintaining ergonomic comfort as compared to traditional static designs.

**Methods:**

Two insoles were customised for 15 children (8–10 years) with flexible flatfeet: a static-based insole (SI), modelled during seated great toe dorsiflexion, and a dynamic-based insole (DI), modelled from the active arch profile during walking. Assessments included geometric characteristics (arch height and medial longitudinal arch [MLA] angle), rearfoot angle (RFA), peak plantar pressure (PPP), and subjective ergonomic comfort.

**Results:**

DI exhibited a significantly greater arch height and MLA angle than SI. Kinematically, both designs reduced severe RFA misalignments, with DI demonstrating more consistent bilateral kinematic control. Kinetically, both functioned as effective total-contact devices, attenuating pressure peaks at the heel and lateral forefoot. Notably, despite the more pronounced structural contour of DI, participants reported high subjective comfort scores that were statistically equivalent to SI.

**Conclusion:**

Both SI and DI effectively improved rearfoot eversion and reduced localized pressures without compromising subjective comfort. Specifically, the dynamic approach achieved greater total-contact load redistribution across the MLA.

## 1 Introduction

Flexible flatfoot is a common structural condition among children during their first decade of life. While flatfoot affects approximately 10%–20% of the adult population, up to 90% of the paediatric population presents with a partial or complete collapse of the medial longitudinal arch (MLA) during weight-bearing (Hameed et al., 2020; Pinzur et al., 2008; Rodriguez and Volpe, 2010). Although many of these cases remain asymptomatic, they are usually accompanied by a series of multi-segmental kinematic and kinetic deviations. Kinematically, flatfoot is most notably characterized by increased hindfoot eversion, commonly defined as a rearfoot angle (RFA) greater than 4° (Rose and Martorana, 2011). Kinetically, affected individuals frequently exhibit abnormal plantar pressure concentrations at the midfoot, heel and metatarsal heads (Xu et al., 2022). Furthermore, the flattening of the MLA and excessive hindfoot eversion disrupt the child’s base of support, exerting strain on supportive ligaments and further weakening key stabilizing tendons (Chinpeerasathian et al., 2024). In many children, the physiological variation of foot development of asymptomatic flexible flatfoot might resolve spontaneously with skeletal maturity without causing functional impairment (Tedeschi, 2024). Consequently, universal orthotic intervention is not clinically indicated for all asymptomatic cases. However, in subsets of children presenting with persistent arch collapse and excessive hindfoot eversion, abnormal kinetic chain loading can disrupt lower-limb postural stability and increase localized tissue stresses during active locomotion (Marouvo et al., 2021; Takabayashi et al., 2021). In such instances, orthotic support is used to optimize walking ergonomics and prevent fatigue. Effective structural control without restricting natural foot kinematics or causing discomfort should also be ensured (Tedeschi, 2024; Fathimahhayati et al., 2025).

Restoring normal foot function and providing structural stability to the collapsed MLA have long been the primary objectives of customized orthoses (Fathimahhayati et al., 2025; Naz et al., 2025). Through specific design features such as medial arch support and posting, these insoles improve overall foot-ankle alignment and reduce excessive hindfoot eversion (Park et al., 2025; Peng et al., 2022). Beyond static alignment, such corrections could also optimize dynamic gait mechanics by shifting loads away from localized peaks and facilitating a more uniform force transfer during the propulsive phase of walking (Xu et al., 2019). However, despite advances in orthotic manufacturing, a major confounding factor in optimal insole design remains the discrepancy between foot behaviour at rest versus during dynamic locomotion. Traditional static impression methods capture the arch in a stationary state, which may fail to reflect the complex and time-varying kinematics of the foot. Recent work highlights that arch behaviour differs significantly between static and dynamic conditions, suggesting that orthoses derived exclusively from static profiles may inadvertently restrict natural foot motion and compromise user comfort during gait (Shiroshita, 2024; Sichting and Ebrecht, 2021).

The advent of highly accurate four-dimensional (4D) scanning systems allows for the capture of foot morphology under functional loading throughout the gait cycle (Allan et al., 2023; Boppana and Anderson, 2021; Sichting et al., 2020; Tajdari et al., 2022; Van den Herrewegen et al., 2014; Zhang et al., 2023). This technology has greatly expanded the capability to contour insoles directly from dynamic arch profiles recorded at specific phases of the gait cycle. Recent advances in additive manufacturing have also revolutionized custom orthotic design, enabling data-driven interfaces to adaptively manage localized stress distribution and conform to complex dynamic plantar geometries (Cracknell et al., 2024). Building on these insights, this study establishes a novel translational workflow by converting active 4D plantar surface profiles captured during terminal stance into customized flatfoot insoles and aims to evaluate the immediate biomechanical and ergonomic effects of these dynamically modelled insoles against traditional insoles modelled from static arch profiles in children with asymptomatic flexible flatfoot. Our primary outcomes were RFA correction and regional peak plantar pressure (PPP) redistribution during walking, while secondary outcomes were insole geometric descriptors and subjective perceptions of comfort and stability. It is hypothesized that while both static-based insoles (SI) and dynamic-based insoles (DI) would reduce RFA and attenuate PPP compared to Baseline, DI would provide a better MLA total-contact and load redistribution during gait; and despite great arch elevation in DI, subjective comfort might remain comparable to SI.

## 2 Materials and methods

### 2.1 Participants

Fifteen right-foot dominant Chinese children (10 boys, 5 girls) aged between 8 and 10 years old were recruited for the study. Inclusion criteria required a diagnosis of flexible flat feet with a standing RFA equals to or greater than 4°. All participants were asymptomatic, reporting no pain or fatigue associated with their foot posture. Participants with a history of lower limb surgery, recent trauma, or neurological disorders were excluded. Written informed consent was obtained from the parents or guardians of all participants, and the experimental protocol was approved by the Hong Kong Polytechnic University Institutional Review Board under the reference no. HSEARS20240125006. Demographic information including the age, body mass, body height, body mass index (BMI), and foot sizes was summarized in Table 1.

**TABLE 1:** Demographic characteristics of children participants (n = 15).

Anthropometrics	Min	Max	Mean (SD)
Age (yr)	8	10	9.5 (0.7)
Weight (kg)	21.2	50.5	36.8 (7.9)
Height (cm)	130	159	141.3 (7.1)
BMI (kg/m^2^)	12.4	23.4	18.3 (3.3)
Foot size (EU)	34	40	37.4 (1.8)

### 2.2 Experimental workflow

The study was structured into three stages: data acquisition, digital design and manufacturing, and a comparative evaluation encompassing biomechanical and subjective ergonomic metrics.

#### 2.2.1 Data acquisition

Foot morphology was captured using a 4D foot scanning system (3dMD, LLC, AG, United States) capable of achieving an accuracy of 0.7 mm. The arch profiles were recorded under two distinct conditions. For the static condition, participants placed one foot on the system’s central mirror (42 cm in length, 19 cm in width) while seated. The operator passively dorsiflexed the hallux to elevate the MLA through its available range of motion (Sichting and Ebrecht, 2021; Kappel-Bargas et al., 1998; Shinohara et al., 2025). For the dynamic condition, participants walked barefoot at a self-selected speed across the scanner’s walkway. The system recorded 3D surface topography at a frame rate of 40 Hz (Zhang et al., 2023). A total of three successful walking trials were captured per participant. The trial closest to the participants’ reference walking speed (±5% variance) was selected for analysis. A single optimal frame from the complete gait cycle was selected as the design template for the DI. This frame was extracted during terminal stance (approximately 31%–50% of the gait cycle, from heel rise of the supporting limb to initial floor contact of the contralateral limb) (Shiroshita, 2024). Specifically, the frame captured the precise moment when the windlass mechanism activated, as the heel lifted and the great toe dorsiflexed to restore the arch profile.

#### 2.2.2 Insole digital design and manufacturing

The three-dimensional (3D) morphological data from both conditions were imported into EasyCAD 2.0 software (Sensor Medica, Rome, Italy) to generate a static-based insole and a dynamic-based insole for each participant. The digital workflow commenced with the identification of key anatomical landmarks: the first metatarsal head (MTH1), the fifth metatarsal head (MTH5), and the heel center. Based on these landmarks, a smooth interpolated 3D surface was generated in proportion to the participant’s foot size to serve as the foundational plantar contact interface. Design parameters were then standardized, applying a general base thickness of 4 mm and a heel cup depth ranging from 27 to 30 mm, appropriately scaled for the participant. The finalized CAD models were fabricated using a fused filament manufacturing 3D printer (EasySnap 3D, Sensor Medica, Rome, Italy) equipped with a 0.8 mm nozzle. The insoles were printed utilizing 85A thermoplastic polyurethane (TPU) (1.75 ± 0.05 mm diameter filament) at an extrusion temperature between 240 °C and 265 °C (Maharana et al., 2022). The design and manufacturing process of SI and DI were illustrated in Figure 1.

**FIGURE 1:**
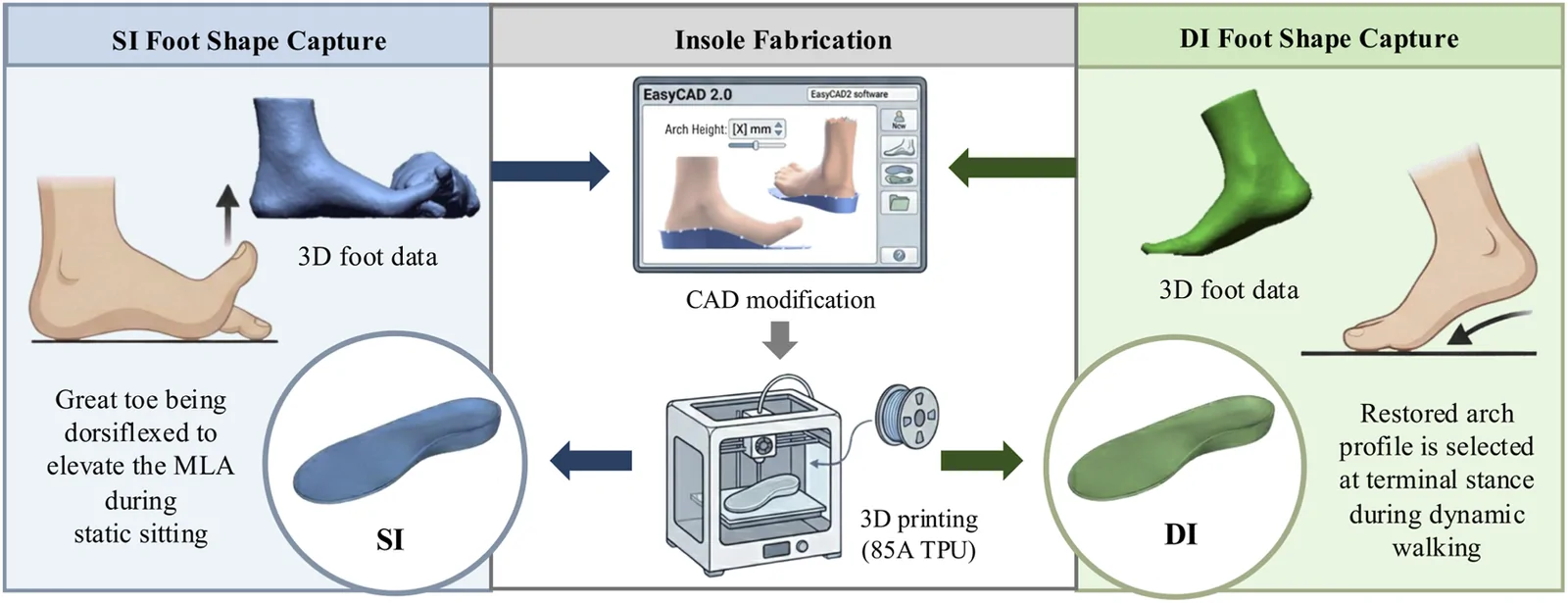
Design and manufacturing process of static-based insoles (SI) and dynamic-based insoles (DI).

#### 2.2.3 Comparative analysis and evaluation

Experiments were conducted across three randomized conditions to eliminate learning effects or fatigue: a Baseline condition, wearing the SI and wearing the DI. Subjective feedback of perceived wearing comfort, postural stability, insole stiffness and arch support were evaluated on a 10 cm Visual Analogue Scale (VAS), which was a well-established comfort assessment tool in footwear and foot orthotics with high reliability and stability (Mills et al., 2010; Mündermann et al., 2002). For the objective assessments, SI and DI were evaluated in terms of their arch profiles, corrective effects on RFA, and peak plantar pressure during dynamic walking, as illustrated in Figure 2.

**FIGURE 2:**
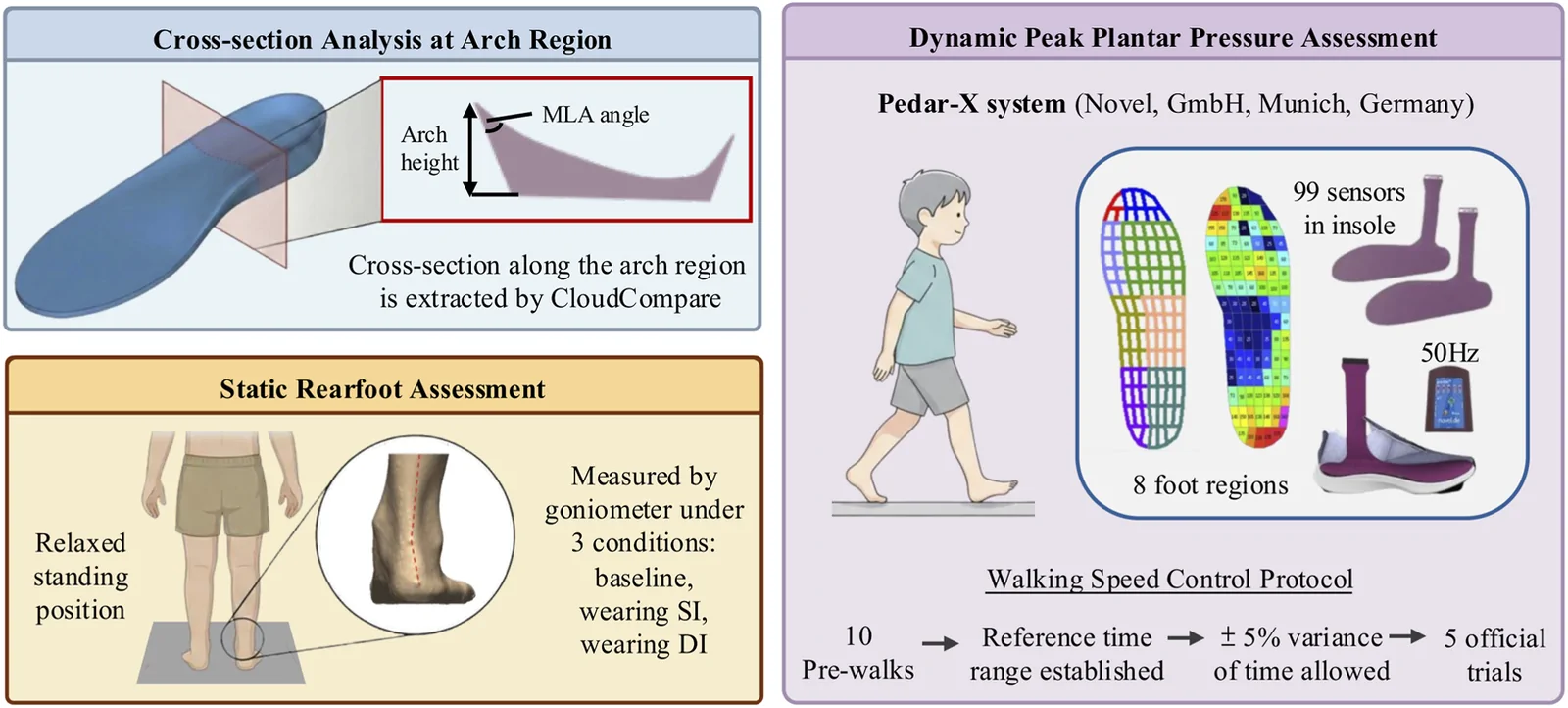
Experimental protocols of evaluating static-based insole (SI) and dynamic-based insole (DI) in terms of arch profile, the corrective effect on RFA and the peak plantar pressure in dynamic walking.

Digital models of the SI and DI were compared using CloudCompare software (version 2.13.2) to analyze the arch height and the MLA angle by extracting cross-sections throughout the arch region along the frontal plane of the insoles. Arch height was defined as the perpendicular distance between the insole’s plantar plane and the highest point of the MLA while the MLA angle characterized the structural elevation of the medial arch. Frontal plane RFA was measured utilizing a manual goniometer (Hill et al., 2013; Su et al., 2017). One experienced examiner performed all goniometric assessments to avoid inter-rater variation (Van Gheluwe et al., 2002). Study-specific intra rater reliability was not assessed.

PPP (kPa) was recorded at 50 Hz using the Pedar-X in-shoe sensor system (Novel GmbH, Munich, Germany). The testing conditions were strictly controlled to account for footwear variables. For the Baseline (sock-bound sensor) condition, the sensor was placed directly against the plantar surface of the foot and secured with a standardized, commercially available sock. For the in-shoe (SI and DI) conditions, the respective insoles were inserted into a standardized sports shoe, and the Pedar-X sensor was placed directly on top of the insole. Participants wore the shoe without a sock to ensure direct interaction between the foot and the sensor interface. To control for the confounding effect of velocity on plantar pressure, a reference walking was first established for each participant by averaging 10 practice trials. At official data collection, participants walked at a controlled, self-selected mean speed that remained consistent across conditions (Baseline: 1.10 ± 0.12 m/s; SI: 1.08 ± 0.07 m/s; DI: 1.09 ± 0.07 m/s) on a 6-m walkway. Five successful walking trials were recorded per condition. Trials falling outside a ±5% variance of the reference time were discarded and repeated. The plantar surface of the foot was mapped into 8 distinct regions for further analysis: hallux, lateral toes, medial forefoot, lateral forefoot, MLA, lateral midfoot, medial heel, and lateral heel.

### 2.3 Statistical analysis

Data were analyzed using IBM SPSS Statistics (version 29.0.2.0, IBM Corp., Armonk, NY, United States). To account for biomechanical asymmetry, left and right foot data were analysed independently. Paired-samples t-tests were utilized to compare the MLA angle and arch height between SI and DI. To evaluate the effect on RFA and PPP across the eight plantar regions, one-way repeated-measures analyses of variance (ANOVA) were conducted with three experimental conditions (Baseline, SI, DI). Significant effects were further investigated using Bonferroni-adjusted *post hoc* pairwise comparisons. Effect sizes were reported using Cohen’s d and partial eta squared ( $n_{p}^{2}$ ) to quantify the magnitude of observed differences. Statistical significance was set at α = 0.05.

## 3 Results

The geometric characteristics of the SI and DI were compared using paired-sample t-tests to evaluate differences in the MLA angle and arch height. As illustrated in Figure 3, the DI exhibited different geometric profiles compared to the SI. SI was generally flatter, providing more space for the foot to flatten and spread, whereas DI featured a steeper medial arch structure and was narrower width-wise, restricting excessive foot motion to a greater extent. For the left foot, the DI demonstrated a significantly greater MLA angle (mean difference = 13.85°, SD = 11.39) and a higher arch height (mean difference = 10.70 mm, SD = 3.78) compared to the SI (p < 0.001, d = 1.22 and p < 0.001, d = 2.83, respectively). A consistent pattern was observed on the right foot, where both the MLA angle (mean difference = 14.36°, SD = 11.04) and arch height (mean difference = 8.87 mm, SD = 3.95) were significantly greater with the DI than with the SI (p < 0.001, d = 1.30 and p < 0.001, d = 2.25, respectively).

**FIGURE 3:**
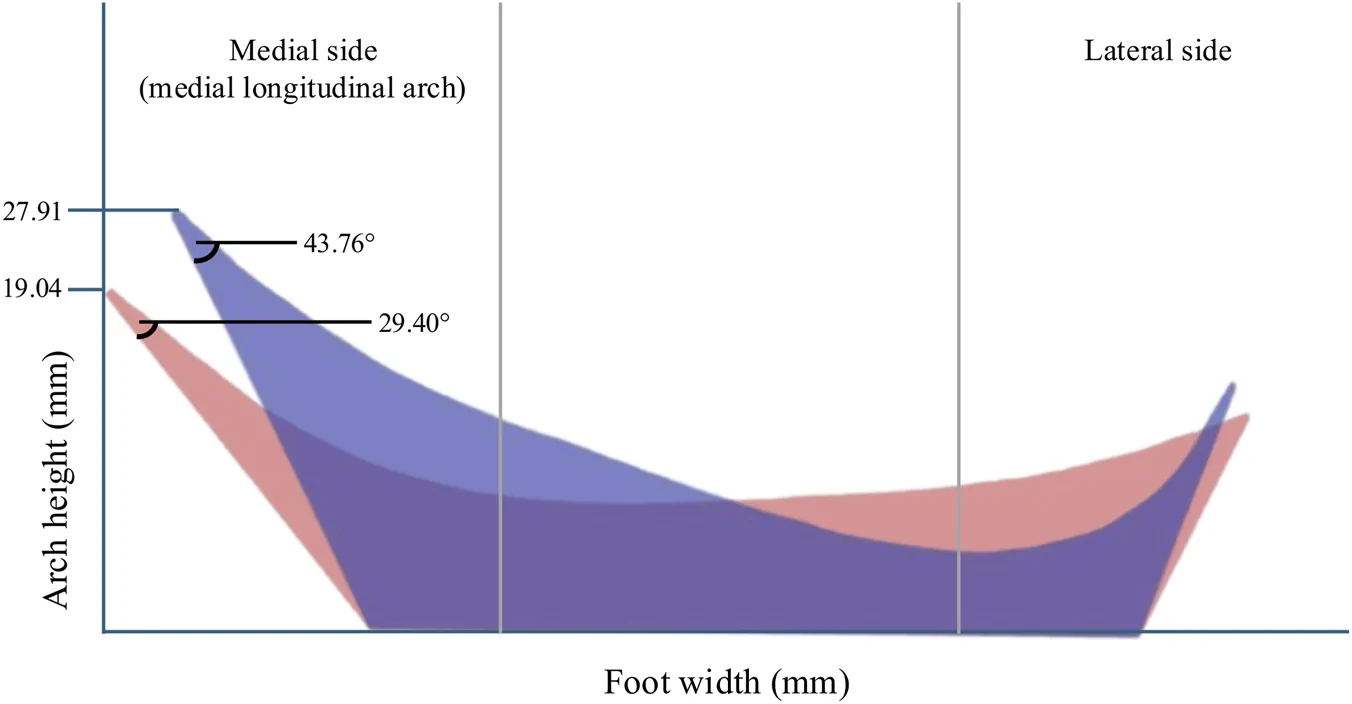
A sample cross-section of the right arch profile in SI (pink) and DI (blue) with the mean MLA height and angle.

Descriptive statistics of RFA across all conditions were summarized in Table 2. A one-way repeated-measures ANOVA revealed a statistically significant effect of the insoles on the RFA for both the left (p = 0.001, $n_{p}^{2}$ = 0.388) and right (p < 0.001, $n_{p}^{2}$ = 0.582) feet. At Baseline, mean RFA was 5.8° (left) and 8.7° (right). Planned pairwise comparisons utilizing the Bonferroni adjustment evaluated specific corrective effects. On the more severely misaligned right foot, both SI (to 6.5°, p = 0.002, d = 1.14) and DI (to 5.8°, p = 0.001, d = 1.30) significantly reduced RFA compared to the Baseline condition, with no significant difference between them (p = 0.227, d = 0.50). On the left foot, DI facilitated a significant reduction to 3.8° as compared to Baseline (p < 0.001, d = 1.37), whereas SI revealed a non-significant reduction to 4.8° (p = 0.302, d = 0.45). Similar to the right foot, no significant difference was observed on the left foot between SI and DI (p = 0.139, d = 0.56).

**TABLE 2:** Descriptive and comparative statistics for RFA(°).

Foot	Condition	Min	Max	Mean (SD)	Comparison	p-value
Left	Baseline	4	12	5.8 (2.8)	Baseline vs. SI	0.302
	SI	0	10	4.8 (2.9)	Baseline vs. DI	<0.001
	DI	0	8	3.8 (2.5)	SI vs. DI	0.139
Right	Baseline	6	14	8.7 (2.3)	Baseline vs. SI	0.002
	SI	2	10	6.5 (2.4)	Baseline vs. DI	0.001
	DI	2	12	5.8 (2.7)	SI vs. DI	0.227

Significant at p < .05. RFA: rearfoot angle; SD: standard deviation; SI: static-based insole; and DI: dynamic-based insole.

Table 3 compared the peak pressure of eight plantar regions across three conditions. PPP concentrated heavily at the forefoot and lateral heel in the Baseline condition. The use of SI and DI created a more evenly distributed pressure profile by significantly attenuating these localized peaks (Figure 4). Specifically, PPP at the lateral forefoot significantly decreased across both feet (Right: p < 0.001, $n_{p}^{2}$ = 0.693; Left: p < 0.001, $n_{p}^{2}$ = 0.710). Similarly, at the lateral heel, the recorded pressure for both insoles was 30%–40% lower than Baseline (Right: p < 0.001, $n_{p}^{2}$ = 0.543; Left: p = 0.038, $n_{p}^{2}$ = 0.258). Within the forefoot, SI distributed PPP relatively evenly between the medial and lateral areas, whereas DI loaded the medial forefoot (Right: p = 0.003, $n_{p}^{2}$ = 0.407; Left: p = 0.034, $n_{p}^{2}$ = 0.265) more heavily than the lateral forefoot. Noticeably, pressure was significantly redirected to the MLA (Right: p < 0.001, $n_{p}^{2}$ = 0.631; Left: p = 0.002, $n_{p}^{2}$ = 0.423). Both insoles recorded MLA pressures 3–4 times higher than Baseline, demonstrating effective total-contact functionality. Recorded the highest pressure in this region, DI seemed to provide a closer match to the foot during walking.

**TABLE 3:** Mean (SD) values of peak plantar pressure (PPP, kPa) and repeated-measures ANOVA across three experimental conditions in eight predefined foot regions.

Foot	Foot region	Baseline	SI	DI	ANOVA F-value	ANOVA p-value	Effect size ( $n_{p}^{2}$ )
Right	Hallux	80.1 (62.8)	150.2 (53.9)^a^	189.9 (66.7)^b^	8.964	0.001	0.449
	Lateral toes	40.4 (13.6)	83.1 (31.4)^a^	86.3 (34.1)^b^	8.885	0.001	0.447
	Medial forefoot	172.2 (58.8)	120.1 (40.8)^a^	151.8 (40.9)^c^	7.546	0.003	0.407
	Lateral forefoot	209.2 (62.4)	115.9 (29.1)^a^	124.9 (35.8)^b^	24.783	<0.001	0.693
	MLA	10.5 (14.2)	34.0 (15.6)^a^	46.6 (14.1)^b^ ^,^ ^c^	18.792	<0.001	0.631
	Lateral midfoot	60.5 (29.2)	85.2 (38.4)	54.3 (14.9)^c^	3.435	0.050	0.238
	Medial heel	209.0 (103.5)	138.1 (26.9)^a^	135.4 (21.6)^b^	7.842	0.014	0.416
	Lateral heel	258.3 (86.8)	179.9 (70.6)^a^	153.9 (55.7)^b^	13.071	<0.001	0.543
Left	Hallux	127.8 (73.6)	164.5 (57.9)	177.5 (67.1)^b^	3.201	0.060	0.225
	Lateral toes	46.6 (27.0)	77.3 (22.4)^a^	63.8 (30.0)	3.721	0.041	0.253
	Medial forefoot	175.3 (77.4)	119.9 (39.4)^a^	167.0 (42.7)^c^	3.960	0.034	0.265
	Lateral forefoot	213.9 (61.5)	118.9 (29.7)^a^	126.9 (34.9)^b^	26.914	<0.001	0.710
	MLA	12.9 (17.6)	37.4 (18.1)^a^	40.4 (14.2)^b^	8.074	0.002	0.423
	Lateral midfoot	57.4 (33.1)	73.9 (24.9)	60.6 (16.0)	0.440	0.650	0.038
	Medial heel	256.6 (75.4)	137.4 (27.4)^a^	128.0 (27.7)^b^	24.474	<0.001	0.690
	Lateral heel	267.6 (77.7)	186.3 (98.3)^a^	170.5 (99.5)^b^	3.822	0.038	0.258

PPP: peak plantar pressure; MLA: medial longitudinal arch; SI: static-based insole; DI: dynamic-based insole. Bonferroni-adjusted *post hoc* pairwise comparisons ( p<.05 ).

^a^ Significant difference between Baseline vs. SI.

^b^ Significant difference between Baseline vs. DI.

^c^ Significant difference between SI, vs. DI.

**FIGURE 4:**
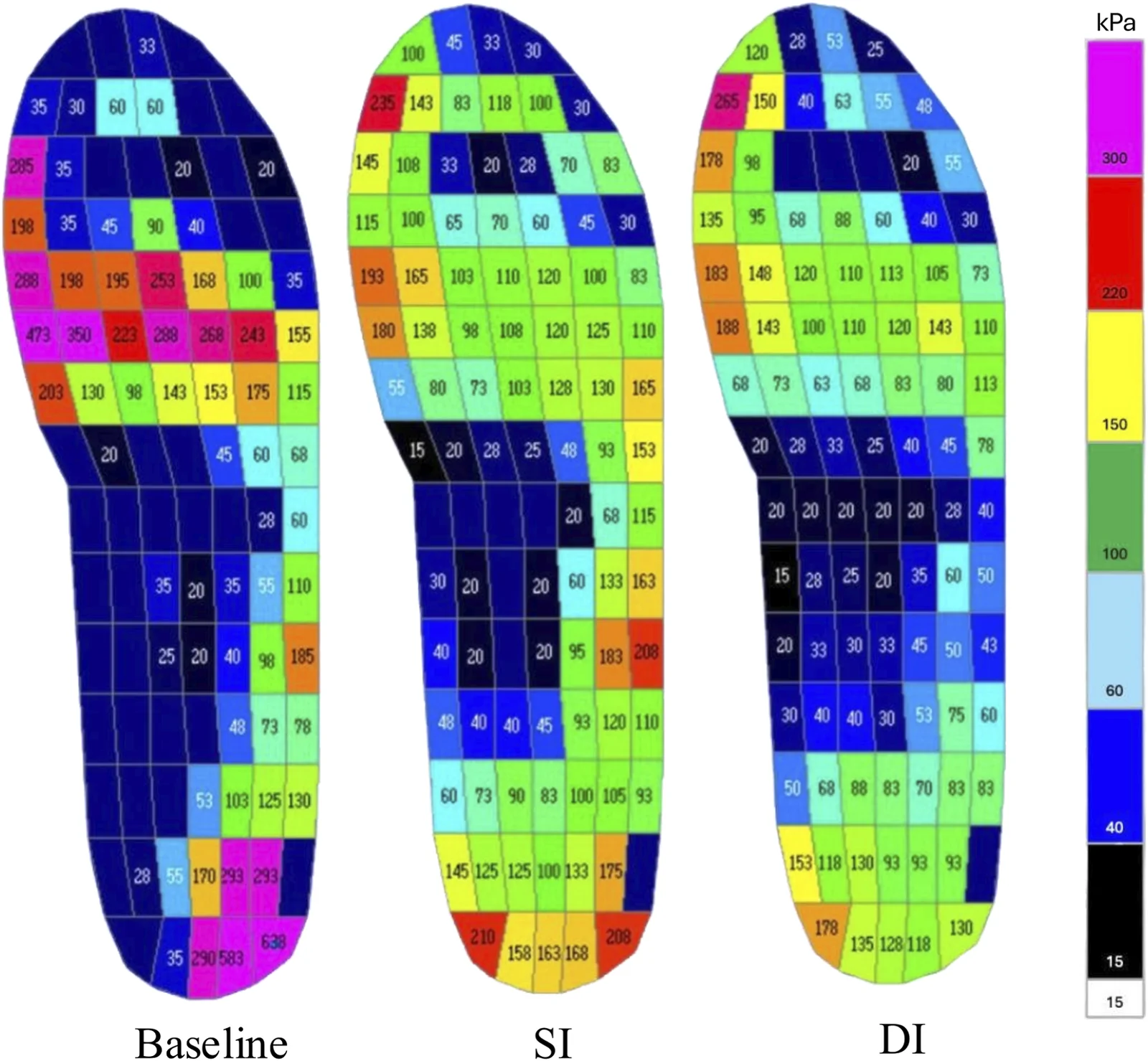
A sample comparison of plantar pressure map across three conditions.

Subjective feedback collected from VAS revealed no significant differences between SI and DI across all four measured domains. Participants reported comparable scores for general perceived comfort (SI: 7.7 ± 1.6; DI: 7.5 ± 1.7; t(14) = 0.581, p > 0.05, d = 0.15), postural stability (SI: 9.0 ± 1.3; DI: 9.0 ± 0.9; t(14) = −0.084, p > 0.05, d = 0.02), insole stiffness (SI: 8.8 ± 1.3; DI: 9.1 ± 1.2; t(14) = −0.915, p > 0.05, d = 0.24) and arch support (SI: 8.7 ± 1.5; DI: 8.2 ± 1.7; t(14) = 1.234, p > 0.05, d = 0.32).

## 4 Discussion

The present study evaluated the biomechanical and ergonomic efficacy of a dynamically modelled flatfoot insole compared to a traditional statically modelled flatfoot insole. The findings revealed that both insole designs improved rearfoot alignments and ensured kinematic efficiency when compared to Baseline. Noticeably, designing flatfoot insoles with active foot morphology during walking gait significantly enhanced dynamic MLA contact and redistributed plantar pressure without compromising subjective wearer comfort.

Extending the great toe in a seated posture elevated the MLA profile. The upward force induced by passively lifting the great toe increased tension in the plantar aponeurosis and pulled the heel toward the metatarsal heads, thereby restoring the height of the arch (Sichting and Ebrecht, 2021). Although SI increased the height of the MLA, inconsistent corrective effects were produced on RFA across the limbs where hindfoot eversion was improved on the right but not the left foot. (Uhan et al., 2023) emphasized that the windlass mechanism should oppose body-weight-induced pronation. When first metatarsophalangeal joint dorsiflexion was performed in a non-weight bearing position, the plantar aponeurosis showed minimal resistance, thereby potentially limiting the translational benefit to weight-bearing function (Long et al., 2025). Overlooking body-weight loading during insole modification may therefore compromise insole efficacy.

The DI was designed to meet the functional demands of gait. Drawing on the analysis in (Shiroshita, 2024) of the MLA height throughout the gait cycle, we selected the terminal stance profile–when the height of the arch was 20.6 ± 6.0 mm, or approximately 49% of the gait cycle—as the design template, rather than the pre-swing maximum (26.8 ± 4.8 mm at 62% of the gait cycle). The rationale was to avoid matching the maximal height of the arch, which might interfere with the natural windlass-driven stiffening that occurred immediately before propulsion. However, our fabricated DIs exhibited mean arch height of approximately 27.7–28.0 mm, which closely approximated the pre-swing maximum rather than the intended terminal-stance value. One possible reason for this discrepancy may lie in age-related differences between the adult population in Shiroshita (Shiroshita, 2024) and paediatric population in the present study. The feet of children typically exhibited greater ligamentous laxity, softer plantar tissues, and more compliant arches (Mickle et al., 2006; Villarroya et al., 2009). It was therefore possible that the arch height observed in adults at terminal stance and pre-swing cannot be compared to those of children with flexible flatfeet. This interpretation remained speculative and would require direct comparison of paediatric and adult arch kinematics to be confirmed.

One of the central findings of this study was the distinct biomechanical divergence between two insole designs. Despite the absence of statistically significant differences between SI and DI, the latter produced slightly larger average reductions in RFA. Compared with the Baseline measurements, DI reduced the rearfoot eversion of the right foot by 2.9° and the left foot by 2°, whereas SI reduced the rearfoot eversion of the right foot by 2.2° and the left foot by 1°. These findings highlighted the value of customized insoles for improving the foot alignment of children with flexible flatfeet. As a result of a higher arch height, a larger MLA angle and a narrower base width, it was assumed that DI constrained the foot and helped limit arch collapse (Kido et al., 2014; Thong-On and Harutaichun, 2023).

Beyond the comparative evaluation in RFA, kinetic analysis also demonstrated that both SI and DI altered the uneven pressure distribution observed in the Baseline condition. In paediatric flexible flatfoot, ligamentous laxity and osseous immaturity often lead to the collapse of the MLA during weight-bearing. It diminished the foot’s natural shock absorbing capacity, leading to concentrated load at the heel and lateral forefoot, a pattern clearly reflected in our Baseline data. Current literature indicated that unmanaged overpronation and poor load distribution can potentially cause biomechanical deviations, such as internal tibial rotation and increased patellofemoral joint stress (Conley and Davenport II, 2018). The introduction of flatfoot insoles thus effectively mitigated these localized peak pressures. Conforming closely to the plantar surface, both SI and DI functioned as effective total-contact devices. By expanding the weight-bearing contact area under the collapsed midfoot, these insoles probably redistributed the forces away from the lateral columns. Research on total-contact interventions suggested that this kinetic offloading was vital for protecting developing paediatric musculoskeletal structures from structural fatigue and repetitive micro trauma (Tedeschi, 2024; Syaifuddin et al., 2025). And it seemed that the dynamically derived arch profile presented in DI appeared to optimize this load transfer. In alignment with recent findings on advanced additive manufactured and dynamically modelled orthotic interfaces (Cracknell et al., 2024), matching the active terminal-stance morphology of the foot, DI improved functional support where a mean pressure of 46.6 kPa (right) and 40.4 kPa (left) at the MLA region was recorded. This simultaneously maintained a more natural pressure gradient and help prevent the structural midfoot crowding occasionally induced by traditional static prescriptions.

In paediatric flatfoot interventions, soft tissue impingement and localized discomfort induced from aggressive structural contour, such as the higher arch and steeper MLA angle observed in DI, were usually the reasons of device rejection (Bai et al., 2025). However, participants in this study reported high and statistically equivalent scores (≥7.0) for perceived comfort, postural stability, material stiffness and arch support across both insole designs. Recent studies analyzing the relationship between plantar pressure and subjective perception suggested that comfort was closely correlated with the avoidance of focal pressure peaks and the congruency of the foot-orthosis interface (Menez et al., 2020). The high tolerance observed in DI might suggest that when the orthotic arch profile aligned with the spatial and volumetric requirements of the foot in motion, the wearer’s tolerance for an aggressive arch support increased. The balance between maintaining corrective geometries and not compromising the subjective comfort could be achieved by dynamic insole design.

Several limitations should be noted for this study. The small and homogeneous sample (N = 15, right-foot dominant children aged 8–10 years old) limited generalizability across subjects of other ethnicities, ages, or limb-dominance patterns. However, the observed limb-specific variance reinforced the needs of independent bilateral reporting in paediatric flatfoot research, as windlass mechanism can vary asymmetrically (Kappel-Bargas et al., 1998). Assessments were also restricted to regional PPP, static RFA and single-plane geometric descriptors (arch height and MLA angle). RFA measurements were performed by one examiner, but intra-rater reliability was not tested in this study. Measurement consistency across repeated assessments therefore cannot be quantified and goniometric measurement error may have influenced the RFA results. Crucially, the present study was designed as an immediate laboratory comparison. Customized insoles often require a longitudinal adaptation period to observe the changes in, for instance, plantar tissue tolerance, muscle activation patterns, and gait kinematics after weeks or months of wearing. Future research should not only incorporate 3D foot kinematics, joint moments or electromyography to evaluate neuromuscular adaptations comprehensively, but also include prospective randomized trials with extended wear protocols to evaluate long-term wearing compliance, structural adaptation and sustained symptom prevention.

## 5 Conclusion

This study compared two customized paediatric flatfoot insoles: a static insole (SI) designed from non-weight-bearing arch profiles, and a dynamic insole (DI) derived from active gait profiles. Both designs provided precise, bilateral kinematic control of the rearfoot and achieved total-contact pressure redistribution. Importantly, both designs maintained necessary biomechanical support without compromising perceived wearer comfort. However, while structural rearfoot alignment was comparable between the two insole designs, DI demonstrated enhanced total-contact functionality, offering significantly greater load redistribution across the MLA of the right foot during walking. Ultimately, this dynamic approach represented a critical advancement, proving that rigid, highly corrective paediatric orthoses can be successfully implemented without sacrificing the subjective comfort required for long-term clinical compliance.

## Data Availability

The raw data supporting the conclusions of this article will be made available by the authors, without undue reservation.
